# Amazonian Medicine and the Psychedelic Revival: Considering the “Dieta”

**DOI:** 10.3389/fphar.2021.639124

**Published:** 2021-05-28

**Authors:** David M. O’Shaughnessy, Ilana Berlowitz

**Affiliations:** ^1^ Department of Psychology, University of California, Berkeley, Berkeley, CA, United States; ^2^ Faculty of Medicine, University of Zurich, Zurich, Switzerland; ^3^ Department of Psychology, University of Fribourg, Fribourg, Switzerland

**Keywords:** diet, dieta, ayahuasca, ritual, psychedelic, traditional medicine, vegetalismo

## Abstract

**Background:** In Peruvian Amazonian medicine, plant diets (*dietas*) are a fundamental and highly flexible technique with a variety of uses: from treating and preventing illness, to increasing strength and resilience, to rites of passage, to learning even medicine itself. Many of the plants used in diets are psychoactive; for example, one now well-known plant that can be dieted is *Banisteriopsis caapi*—the vine also used in the psychoactive brew *ayahuasca*. The use of ayahuasca has attracted increasing clinical attention towards Amazonian medicine in recent decades, and much work has focused on the potent DMT-containing ayahuasca brew, thus placing the tradition within the purview of psychedelic science.

**Aims:** In comparison to ayahuasca, the properties of diets have been studied less often. Our work draws on data from Amazonian healers to examine plant diets as medical practices, while also considering their fit within the “set and setting framework” that is central to psychedelic research. We argue that the framework is not sufficiently broad for understanding diets, and thus the investigation aimed to expand the conceptual field of Amazonian medicine, particularly in the context of a renewed psychedelic science and its theoretical concepts.

**Design:** We used qualitative data from interviews with Amazonian healers, applying a thematic analysis and contrasting findings with the available literature.

**Setting:** Interviews were conducted in various locations in the San Martín province of Peru between 2015 and 2017.

**Participants:** We selected and interviewed eight healers who had been extensively trained in traditional Amazonian medicine.

**Measures:** Semi-structured interviews were used to gain insight into the healers’ personal experiences with plant diets.

**Conclusions:** Diets are complex but understudied medical practices that should not be explained by reference to pharmacology or psychology only. Intercultural and interdisciplinary research programmes are called for in order to not only better understand plant diets, but traditional Amazonian medicine on the whole.

## 1 Introduction

Thirty years have now passed since the resumption of psychedelic studies with human subjects (Strassman, 1991; Carhart-Harris and Goodwin, 2017), and the field no longer carries its taboo status acquired after the earlier collapse of the research programme and a punitive and decades long “War on Drugs” (Szara, 1967; Dahlberg et al., 1968; Novak, 1997; Sessa, 2005; Morris, 2008; Wood et al., 2009). To the contrary, in the last decade impressive clinical outcomes for a range of conditions (Grob et al., 2011; Murrough et al., 2013; Oehen et al., 2013; Bogenschutz et al., 2015; Carhart-Harris et al., 2016; Johnson et al., 2016; Ross et al., 2016; Roseman et al., 2018; Fuentes et al., 2020) have spurred growing medical and psychiatric interest regarding psychedelic-assisted therapies and the promise they may hold for alleviating human suffering (Tupper et al., 2015; Chi and Gold, 2020).

The utility of mind-altering substances such as the classical hallucinogens is relatively new for Western scientific medicine however, and the compounds pose a unique challenge to the advancement of a purely biomedical or pharmaceutical approach to health. During the first wave of psychedelics research, one aspect of this challenge was understood and expressed via the theoretical constructs of *set* and *setting* (Solomon, 1966; Eisner, 1997; Hartogsohn, 2017). Put simply, the early researchers recognized that the outcome of any psychedelic drug administration was not determined by the drug alone, but rather was heavily dependent on the intentions, expectations, and general psychological state of the person receiving the drug (the set), and crucially also on the immediate environment in which the drug was taken (the setting; e.g., Olson et al., 2020). For this reason both therapeutic context and behaviour assume paramount importance in psychedelic therapy, and clinical protocols have therefore been developed which combine psychotherapeutic intervention with a pharmaceutical approach (Schenberg, 2018).

### 1.1 Indigenous Roots

Despite the novelty of psychedelic-assisted therapy for psychiatry, the use of hallucinogens by indigenous peoples is ancient (Merlin, 2003; El-Seedi et al., 2005; Guerra-Doce, 2015; Miller et al., 2019; Robinson et al., 2020), and thus the application of such plants in the pursuit of healing is not a Western discovery (Solomon, 1966; Schultes, 1987; Grob, 1994). Indeed, it is notable that mainstream psychiatry has not seriously engaged with indigenous and traditional healers who make use of powerful consciousness-altering plants (Albaugh and Anderson, 1974; Pascarosa et al., 1976; Calabrese, 2001), although the therapeutic potentials are now beginning to attract wider attention. But even though psychedelic compounds isolated from plants can in principle be used in therapeutic contexts (just as LSD was used in classical psychedelic or psycholytic therapy), the question of whether the classical theoretical tools that have been developed to explain and guide psychedelic therapy are equally suitable for understanding the rich indigenous practices that precede the current psychedelic medical revival is non-trivial. While the important concepts of set and setting vindicate to some extent the approach of the shamanic healer who utilizes psychoactive plant medicines (e.g., through a pharmacologically modulated cultural/semiotic system; Dow, 1986; Lévi-Strauss, 1986), we argue here that the concepts are not on their own sufficient, and in fact carry the danger of reducing or mistranslating sophisticated medical traditions that make use of consciousness altering plants as arbitrary cases of “set and setting”.

### 1.2 Ayahuasca

One example can be seen in the case of *ayahuasca* (roughly translated from Quechua as “vine of the soul”), a psychoactive plant brew originating in Amazonia with a variety of traditional uses (Schultes, 1982; Luna, 2011). Still used for instance in Colombia, Brazil, or Peru according to local traditions, the brew has also undergone a process of globalization in recent decades, initially through so-called “ayahuasca tourism” at South American ayahuasca retreat centres, but also through increasing religious, neo-shamanic (Scuro and Rodd, 2015), and underground usage throughout the world (Grunwell, 1998; Winkelman, 2005; Dobkin de Rios, 2006; Dobkin de Rios and Rumrrill, 2008a; Tupper, 2009a; Labate and MacRae, 2010; Labate and Feeney, 2012; Cohen, 2014; Labate, 2014; Gearin, 2015; Kavenská and Simonová, 2015; Fotiou, 2016). While in academic and popular use the word “ayahuasca” frequently refers to a decoction of at least two plants—usually a vine containing monoamine-oxidase inhibiting β-carbolines (*Banisteriopsis caapi*) and the leaves of a shrub containing *N*,*N*-dimethyltryptamine (DMT; e.g., *Psychotria viridis*)—the name also refers to the *B. caapi* vine itself (Luna, 2011).

Much like the classical psychedelics, ayahuasca use has been associated with a range of positive therapeutic outcomes in the clinical research literature, including the alleviation of depression, persistent grief, and addictions (Grob et al., 1996; Palladino, 2009; Thomas et al., 2013; Dos Santos et al., 2016; Argento et al., 2019; Giovannetti et al., 2020; González et al., 2020), without evidence of cognitive impairment or psychological maladjustment (Bouso et al., 2012). Even though the historical roots of ayahuasca usage are often acknowledged in medically oriented reviews (Dos Santos et al., 2016; Hamill et al., 2019), the modern tendency to view the ayahuasca vine as primarily a pharmacological vehicle for making DMT orally active (McKenna et al., 1984; Riba et al., 2014) allows for ayahuasca to be more easily situated within a psychedelic framing. While such a framing might allow for a smoother integration with Western medicine, it obscures the diversity of ayahuasca preparations (some of which do not contain DMT) and use in its homelands, as well as the historical and contemporary significance of the vine itself cross-culturally (Schultes, 1982; Schultes, 1987; Rodd, 2002, Rodd, 2008; Spruce, 2005; Luna, 2011; Politi et al., 2020). The special focus on DMT, which may be used in higher concentrations in more contemporary settings (Kaasik et al., 2020), also works to obscure the importance of other plant preparations in the Amazonian healing landscape beyond ayahuasca: In the Peruvian mestizo medical tradition known in some regions as *vegetalismo*, a marked difference can be seen in studies that report on a diverse world of master plant “teachers” capable of curing different ailments while also imparting knowledge to humans (Luna, 1984a; Luna, 1986; Kamppinen, 1988; Jauregui et al., 2011), and contemporary reorientations of the tradition (under conditions of globalization) where ayahuasca is becoming increasingly central (Labate, 2014).

### 1.3 Plant Diets

Within the diversity of the Peruvian vegetalismo tradition, one especially common technique for healing or learning how to heal is the *dieta* (diet). By combining the intake of certain plants along with social isolation, alimentary restrictions, and sexual abstinence (Luna, 1984a; Jauregui et al., 2011), *curanderos* (healers) report that the spirits of those plants will become manifest (either in visions or through dreams and songs), which opens a communication channel and is supposed to make possible a literal learning from nature. According to the curanderos that Luna (1984a) spoke with, plants that can teach will either: “1) produce hallucinations if taken alone; 2) modify in some way the effects of the ayahuasca beverage; 3) produce dizziness; 4) possess strong emetic and/or cathartic properties [or] 5) bring on specially vivid dreams” (p. 140). Combined with the relational concepts of plant-spirit communication (Callicott, 2013; Daly and Shepard, 2019) it becomes evident that such practices draw on a variety of ontologies and epistemologies not well described using a purely psychedelic model. Indeed, complex modes of indigenous thought that relate sickness and health to a greater web of ecological relationality (including relations between humans, plants, animals, and other beings) have been described in the anthropological literature (e.g., Reichel-Dolmatoff, 1976; Wilbert, 1990; Århem, 2004), often predicated on the idea that animals and plants are in essence themselves “people” (De Castro, 1998).

Moreover, in contrast to an explanatory model where mind is paramount, Amazonian conceptions of plant healing typically place special importance on the body. As Fotiou (2017) writes, plants in Amazonia “are not used to change the user’s consciousness but to imbue the body with certain properties”. It is important then to note that plant diets are not contingent upon the use of ayahuasca or other plant hallucinogens at all; they have a wide range of application that is often understood to function through a form of purification, either bodily or spiritually (Sanz-Biset et al., 2008). The focus on bodily purification as an aspect of healing has also been noted in contemporary Amazonian healing practices (Fotiou, 2019), although such action is not easily reducible to biology alone, as it interfaces in complex ways with culture, behaviour, and affective states (Fotiou and Gearin, 2019).

### 1.4 Rationale and Study

Given the scientific currency of psychedelic medicine and its probable convergence with folk technologies like ayahuasca, for example through a standardized “pharmahuasca” approach (Ott, 1999; Geyer and Halberstadt, 2016), it seems imperative not to lose sight of the broader medical traditions within which those technologies have been developed and are embedded, particularly with respect to how they might differ from a psychedelic or biomedical approach to medicine. The aim of the present study then was to demonstrate the ways in which the psychological concepts of set and setting—especially when combined with the reduction of medicinal plants to isolated bioactive compounds—are not sufficiently broad to account for the complexities of Amazonian medicine, and the plant-based medicines and associated practitioner techniques developed within this tradition.

## 2 Methods

### 2.1 Data Collection and Participants

The data reported in this work were collected in the context of a broader qualitative study which aims to describe Amazonian diets as a therapeutic intervention, based on semi-structured interviews with practitioners of Amazonian medicine across the Peruvian Amazon. The data collection of this broader study involved two separate collection stages; namely, “set A” which was collected in the San Martín province of Peru (2015–2017), and “set B” which was collected in the Ucayali and Loreto provinces of Peru (initiated 2019). For the purposes of the current work, we used data from set A (with set B analyses still being in progress) and focused on the section of the interview in which we had asked the healers to describe their personal experience with plant diets they have undertaken. The current analysis aims to highlight specific features of these diet accounts as an Amazonian medical tool in relation to the psychedelic medicine revival and its concepts. It does not aim to provide a comprehensive description of the diet process itself, which will be reported elsewhere (based on the full set of data from the broader study).

The current work’s participants consisted of eight practitioners of Amazonian medicine (data set A). These participants had been identified with the help of the Takiwasi Center, a therapeutic institution in the Peruvian Amazon which has applied Amazonian medicine to treat mental health issues (particularly addictions) since 1992 (Berlowitz et al., 2017; O’Shaughnessy, 2017; Politi et al., 2018), and which possesses an extensive contact network of Amazonian medicine practitioners. The study was approved by the responsible ethics committee (University of Fribourg, Switzerland) and informed consent was obtained from all participants. All eight participants had been trained under the tutelage of one or several traditional Amazonian healers, and all of them had at some point in time served plants at the Takiwasi Center. Seven participants were Peruvian nationals living in the San Martín Province, and one participant was Argentinian. One of the Peruvian participants had a background of European upbringing. These latter two participants were included in the analysis nonetheless, as they were highly experienced practitioners firmly embedded in the Amazonian medical tradition for over 25 years. All participants were extensively schooled in traditional Amazonian healing, reporting teachers and relatives that were healers from various Amazonian traditions (Kichwa-Lamista, Chazutino, Asháninka, Awajún, or mixed Amazonian).

### 2.2 Analysis

All interviews were conducted in Spanish and transcribed verbatim (second author). For analysis, an iterative process of identifying and coding themes and subthemes (first author), and subsequently discussing and refining the themes (both authors) was applied. Quotes were selected to illustrate each theme, which were then translated into English. The names of participants and other identifying information (such as third persons mentioned in interviews, or healer-specific plant mixes) have been removed in the interest of maintaining participant confidentiality and privacy. These English translations have been edited for clarity while avoiding alteration of the expressed meaning in significant ways. Our participants are referred to as P1 through P8.

Along with the presentation of patient quotes and discussion, we also draw on relevant literature, by which we intend to demonstrate the links between past and current practice; that is, highlighting that Amazonian medicine is at once both traditional and contemporary. While each healer may have their own idiosyncratic perspective (as is characteristic of Amazonian and other forms of shamanism), by creating a bridge to the research literature we also aim to show that our selected themes reflect understandings present in Peruvian Amazonian medicine more broadly and are not limited to our specific data and set of interviewees.

## 3 Results and Discussion

From our analyses we extracted the following themes and sub-themes: 1) the diverse applications of diets (including somatic medicine, psychological healing, and plant teachers for learning medicine); 2) conditions of the diet (including rules and fundamental components, and the breaking of rules); and finally 3) diets in relation to the use of ayahuasca. We present each theme with selected quotes from our data, along with literature comparisons where relevant.

### 3.1 Diverse Applications of Diets

The use of plants is central in Amazonian medical traditions. As urban hospitals are often remote and not accessible for indigenous and mestizo communities, the medicinal use of plants can account for treatments across a very broad range of illnesses and health conditions, which points to a long-standing indigenous medicine (Berlowitz et al., 2020; Jauregui et al., 2011; Sanz-Biset and Cañigueral, 2011). For example, an initial community survey of medicinal plants by Sanz-Biset et al. (2008) in the Chazuta^1^ region of Peru revealed 318 different plants with a wide variety of application: from physical problems like infections, stomach pain, lumbago, headaches, scabies, bronchitis, malaria, diarrhoea, toothache, snake bites, broken bones, and wound care, to general health tonics (e.g., for giving vitality), preparations for hunting (applicable to both humans and dogs), rites of passage for adolescents, augmented work performance and reduced laziness, sharpened senses, increased sexual vitality, the attainment of special abilities for shamans, and also the treatment of conditions believed to be caused by sorcery or evil spirits (e.g., *mal de aire/gente*; Kamppinen, 1988; Sanz-Biset and Cañigueral, 2011, Sanz-Biset and Cañigueral, 2013). Lists of medicinal plant usage have been compiled for other areas of Peru (Jovel et al., 1996), including for specific conditions such as malaria and leishmaniasis (Kvist et al., 2006), or senile dementia (Schultes, 1993).

Plant diets, where one remains secluded in the forest for an extended period of time while consuming minimal food in addition to the medicinal plant itself (see next theme cluster), are a fundamental technique in Amazonian plant medicine. Though the early work of Luna (1984a, 1984b), made diets for learning medicine (which we will address later) well known, there are many other extant uses. The wide range of application is a striking feature of diets, and one which was also reflected in our interviews. In what follows we report a number of such uses representing themes that were prevalent in our data (but which should not be taken to represent an exhaustive list).

#### 3.1.1 Somatic Medicine

The majority of diets in Sanz-Biset and Cañigueral’s data (2011) were employed with the aim of healing specific physical conditions, which was also acknowledged in our interview data:

P2: The diet is mainly a process of healing and learning. Healing of mainly physical illnesses, for example rheumatism, women’s problems in the belly, dislocations, snake bites, some physical problem, an illness, accidents. In this case the person may approach a healer, and the healer will give them a plant according to their physical illness. For example, if they have rheumatism, the healer will perhaps give them [lists diet plants], the healer will decide.

Some of our participants described medical applications and indications for specific plants:

P8: [Diet plant 1] is used for cleaning, for memory, stomach ulcers, for washing wounds; it cleanses you well. You cook it with a little bit of salt and use it for washing. Also for parasites. […] I use [diet plant 2] for protection of the lungs, bronchi, to heal asthma. All that cough is stored in the lungs, with pills and medicines it doesn’t improve. When dieting [with diet plant 2] it gets extracted. It extracts [the phlegm]!

Other participants mentioned their own experience with recovery from a somatic problem, for instance a diet with a plant often used as a treatment for rheumatic diseases:

P3: With the [diet plant] I have another experience as well, it took out or cured the cold. I had arthritis or rheumatism, but now I am already 60 years old and since I am taking [diet plant] I have no pain whatsoever. I even went to the hospital due to the rheumatism. And the doctor asked me, “Does someone in your family suffer from rheumatism, your father or mother? Because it is inherited.“, “Yes doctor, my father”. So I have inherited it, but since I am taking these plants, nothing anymore.

People who are otherwise healthy may partake in diets for the purpose of gaining strength (Sanz-Biset and Cañigueral, 2011), which is of broad utility, but such strength giving properties are also believed to act as a preventative against illness or as a direct healing agent for the injured. One informant mentioned a decoction of mixed tree barks often called *palos* ^2^ (another name is *bachuja*; Sanz-Biset and Cañigueral, 2011), noting an ability to impart physical strength, improve posture and bone structure, but also to speed recovery from internal injuries and assist in wound healing:

P6: So palos also heal, they are healers. It helps the person to have strength, mainly that. As they say if someone dominates you with their strength—ah, you are lacking a diet with palos! And this is the effect it has on us, you get injured and take palos, that helps you to heal, it helps you with strength and things like that. It also activates [reduces lethargy], but mainly it is a healing agent—if you suffer from a wound, a woman that has pains in their abdomen, perhaps in their uterus, it helps to heal wounds. This is the effect of palos. My uncle broke his leg once, it wasn’t necessary to operate, you just have to place the bone in the right spot, put on a bandage, and send him off to diet. But it depends on how serious it is, there are some who have surgery and then go on to diet. […] On a physical level it mostly helps you, so that you have good posture standing and sitting, palos helps you with that. That’s why sometimes people with fractures, they diet palos, and it helps. Those are my past experiences, the experiences of my parents, of my brothers.

Finally, while scientific research on diet plants as a whole is relatively slim, it is worth noting that Sanz-Biset and Cañigueral (2011) found quite good concordance in the available literature for the anti-inflammatory and antibacterial properties of diet plants (including related taxa) that were commonly valued for those purposes. For example, compounds isolated from *Maytenus macrocarpa* (*chuchuwasi*) have shown both anti-inflammatory and antibacterial properties (Malaník et al., 2019). However, the range of plants used in diets is very large, and they are valued as a means of treating a wide variety of somatic health conditions, in addition to preventing illness by ensuring general health, vitality, and resilience more globally.

#### 3.1.2 Personal Change and Psychological Healing

In our interviews, diet plants were often discussed as capable of healing maladaptive psychological processes and behaviours, with the plants themselves frequently being attributed agency as guides or teachers: “In diets with master plants, the master plant gives you dreams, it heals the wounds of your bad thoughts, the wounds of your addictions. So it heals you, it guides you, it teaches you, it polishes you—because the master plants heal” (P3). Although our informants were healers themselves, they spoke of having attained their own beneficial changes in terms of personality structure or psychological well-being:

P5: In my town I have taken the remedies, since a young age I have taken them, that’s why I had [knowledge of the plants], I knew how these plants are. […] Let me tell you, I was in the army for two years, in the time of terrorism, that is, I have served my country. And when you’re there in the army—because living the civilian life, that’s something else, it’s more peaceful than living a military life. So you get a little bit, the character gets a little bit hard. I mean, it rubs off on you. And I brought that strong character with me [out of the army]. And with the diets, there [in the chacra],^3^ they have shown me. I had dreams too. And I feel that [my character] changed. I feel that all of these things have changed, and many other things. A huge change. […] In the army everything was always fast [demonstrates shouting], that changed. Now I have patience, more calm.

The idea that diets can be utilized with psychotherapeutic goals in mind was a frequent theme in our interviews. For example, one informant noted an ability for diets to generate an abrupt insight: “There can be effects on a simultaneous psychological-emotional level, it can manifest itself with awareness, of things that one could not previously understand—then, prah! There is an insight, a kind of understanding” (P7). Several participants reported experiences of this nature, where psychological insight of healing was not something explicitly sought out or even expected, but rather seemed to occur as a natural by‐product of the diet process:

P2: That diet was very revealing for me, very important, because while I was expecting a cleansing effect of a spiritual-energetic nature, what happened was a tremendous emotional release. […] At some point in the diet it began to rain, and I had this song in my head, and pum, I associate this event, or it arises, the memory of my sister’s death. And I start to cry, deeply, to the rhythm of the rain I was crying—I was surprised. […] I don’t feel that I have been depressed, but it’s certainly true that I have had a grief that was not processed or digested. When my sister passed away I was younger, in a phase of life as a teenager, trying to be manly, macho, and trying not to show fragility or weakness. When she passed away, for some reason I didn’t process the emotion, I don’t remember having cried or mourned. That is, [the emotion] got blocked and came to the surface only 20 years later on that diet.

This theme of early life content arising in an unexpected manner during a diet was echoed by another participant:

P1: A lot of information, a lot of awareness so to speak. I don’t know if it is related to the training to become a healer, but I feel it is like knowledge that has been in the body and from one moment to the next you become aware of it. It’s not that it comes from another place, it has already been there and one simply didn’t notice. My first diets basically involved working through childhood stories, linked to my mother, including some things I’ve experienced in my childhood, which in reality I didn’t feel to be traumatic. I never thought that I had suffered as a child, that I had been traumatized—absolutely not. And yet in that diet things came out that I didn’t know I had stored inside and that would perhaps impact me somehow internally […] when I talked with my mom, it turned out that these indeed were actual situations that had happened in reality.

In the context of therapeutic centres offering Amazonian diets (such as Takiwasi and others) the effects or aspects of the plants that may offer benefits in the psychological domain can take on greater significance, as attested by another informant: “The notion of a diet as a retreat, aimed to think about your psychological or personal problems, or like in the case of Takiwasi reflecting about an addiction, that is more or less a recent notion” (P2). However, this shift in focus towards psychological introspection for therapeutic centres does not imply that those benefits are absent in the indigenous and remote community use of diets, or that they are a pure invention of Western‐influenced therapeutic centres. Indeed, given the holistic nature of the Amazonian understanding of health and healing, it is more likely in local community contexts that “psychological benefits” are not partitioned out as a separated health category (Conklin, 1996), where instead gaining strength, energy, and healthy functioning in a global sense already implies a healthy state of both mind and body. Yet regardless of how the psychological component is framed, diets do appear to foster introspection (recalling the social isolation that they are held in), although they tend to be viewed as having multiple types of effects simultaneously, as one informant illustrated:

P4: It depends on the plant. All effects can be on the psychological plane, on the physical plane, or on the spiritual plane as well. On the physical plane, if someone has a specific affliction, arthritis or arthrosis, and the plant works on that plane for example, some cancer, or possibly an immunodeficiency, and on the psychological plane it is an entire process of introspection and of reaching an understanding regarding different psycho-emotional aspects of the self. Revisiting their affective world, relational world, a reconsidering of one’s professional calling, one’s place in the world, what one is doing, what one would like to do, where one wants to direct oneself. And in addition there is also a spiritual plane on which things arise, revelations, contact with the transcendent.

#### 3.1.3 Teachers of Medicine

Aside from their perceived value for physical or mental health benefits, Amazonian diets have another important function which is also medical, but in a radically different way. Namely, it revolves around learning the work of a healer and training in a type of medicine which can be classified as shamanic (Rock and Krippner, 2011; Scuro and Rodd, 2015). In the anthropological literature, the function of diets as a means of learning this kind of plant medicine are well known: Luna’s now classic early publications (Luna, 1984a; Luna, 1984b; Luna, 1986), and later with Peruvian curandero Pablo Amaringo (Luna and Amaringo, 1999), describe a complex and dangerous “invisible world” that becomes accessible to the practitioner, with entry implying exposure to forces both good and evil (Kamppinen, 1988; Harner, 1993; Dobkin de Rios and Rumrrill, 2008a). Given that this function is intended for the specialist, it follows that it remains less commonly reported at the community level when compared with the more direct health and life improvement aims mentioned earlier (Sanz-Biset and Cañigueral, 2011). However, as our sample consisted of traditional Amazonian medicine practitioners, the theme of plants as teachers of medicine in this sense was prominent:

P2: What is interesting about the diet is that many healers have learned to be healers while dieting. That is, while getting treated for a physical illness in the context of this world of plants, the spirits show themselves. Let’s say that the spirit of the plants or their consciousness, or their self healing force—whatever this may be—manifests and tells the person, “You can be a healer”. And the person will have dreams, signs [about this] all the while their physical body is being cured.

It is notable then that personal healing effects are not absent in these kinds of diets, but rather that the learning process and the teachings by the plant spirits are happening concomitantly. Thus diets for learning or for healing are not in fact discrete categories, but overlapping. For example, one of our informants narrated the story of how he fractured his leg in an accident, as an adolescent. Incapacitated by the injury, he was prescribed a lengthy plant diet for the leg to heal, but ended up discovering his potential to become a healer during this process. Examples of such initial healing experiences and subsequent apprenticeship were described by further participants, among them one healer who reported having had a problem (*un vicio*) with alcohol:

P3: When I drank those plants I no longer had any desire for even one beer—no alcohol or cigarettes—my anxiety was gone. Now, apart from that, when I was taking that remedy, that mix, I was rejuvenated, and that gave me some inner strength and an apprenticeship. I already had dreams, I dreamed about [name of teacher], that we were swimming in the river, I was singing with him, I was healing with him, he was going to my house in my dreams. So I was already beginning to learn, I was given teachings and I was also being healed. Imagine that, these plants indeed heal. Because these plants [lists names] rebuild you, they give you strength, they give you an inner strength, they give you teachings, they give you curiosity, they give you motivation.

The idea of simultaneously being healed and learning how to heal was echoed by another informant, who emphasized personal healing along with a body-based form of learning:

P2: The diets for me were more for personal work, and a bit for learning to cure, although this was not being processed by my head, but by my body. The usage of tobacco, the sopladas [blowing of smoke], the songs, all of that gets prepared and refined in the diets. […] The diet offers knowledge that one can remember in the context of one’s personal healing process, but the diet also offers a type of learning that takes place in the body, which does not involve the intellect.

The blowing of tobacco smoke (*soplar*; Berlowitz et al., 2020) described by the previous informant is an extremely common Amazonian shamanic technique^4^ with many functions, including at least medical, ritual, and spiritual—although these categories again are not necessarily discrete, and tend to overlap in a holistic understanding (Stirling, 1933; Kroeber, 1941; Luna, 1984b; Wilbert, 1990; Wilbert, 1991; Bennett, 1992; Sharrock, 2018; Berlowitz et al., 2020). The “singing” above refers to the singing of healing songs (*icaros*) which are held to have their origin in the spirit world of plants, and which are well known for being employed during ayahuasca rituals (Bustos, 2008; Tupper, 2009b; Fotiou, 2012). Receiving icaros through a plant diet featured in our interview data for multiple participants:

P1: With each of these plants I received an icaro. Even with the [diet plant]—although they say that it’s not so visionary—but yes I have had insights. I’ve had lucid dreams, so to speak, very strong, to the point of seeing how they came to bring me the plant medicine: it was a red-headed guy with a hat and a beard, he came humming the song of the [diet plant]. So that’s where that icaro came from.

The receipt of icaros through visions or dreams (Luna, 1984a) points to the shamanic terrain which practitioners often describe in relational terms concerning entities who inhabit otherworldly realms:

P3: In my diets I also learned rituals, to sing as well—I have an icaro from that diet—and they have given me quite a lot of knowledge about rituals. In aerial trains that I have seen in my dream I was taken to a ritual with a maestra, an old lady, she took me but they did not want to let me into the ritual. And the elder, my maestra, she spoke on my behalf and they let me in. An enormous ritual, with maestros from all around the world, and with tremendous octopuses, giant sea octopuses were there. The ritual took place in a huge maloca, and in this maloca there were a lot of good things and a lot of negative things as well. In essence, they took me to that ritual in order for me to learn which ones were good and which ones were bad: what the good ones do and what the bad ones do.

This report is highly reminiscent of the material found in Luna and Amaringo (1999), where modern day symbols and technologies (such as spaceships) co-exist fluidly with natural and mythical creatures inside a complex and dualistic world of ritual and magic. In other cases informants’ diet experiences were more firmly rooted in natural elements, but still featured entities that could impart knowledge:

P1: I first dreamed that I was in a forest. And I was beginning to recognize the plants, and they were the plants from when we had been preparing the remedy. Before that I did not know how these plants looked in their natural physical form, that is I only had seen the pieces of bark that were already cut up, we had brought a costal [type of bag used in the Amazon for carrying heavy loads] filled with chunks of bark, but I had not seen the trees to which they belonged. But in the dream there was a forest and I was passing by and I was saying, “Oh, that’s so-and-so plant”, and suddenly a little door opened at the trunk, and a tiny character came out, from inside the tree, like a duende [elf/leprechaun], earth-coloured, somewhat greenish, I don’t know if he was dressed or naked, but he was small, not a child, but short. And he told me, “Ah, I know how to cure such a thing”, and then he would walk off and sing, show me the twigs. And similarly with each one of the plants. […] And it was a strange thing, because I would wake up and say to myself, “What a silly thing I’m dreaming”; but as soon as I went back to sleep it would continue, because it was thirty-odd plants, so it was a very, very long dream. Each plant came out, opened up, spoke and taught me.

### 3.2 Conditions of the Diet

#### 3.2.1 Rules and Fundamental Components

Our informants nearly universally stressed the importance of maintaining specific dietary and behavioural taboos in order to achieve a therapeutically effective and safe process. These taboos centered on social isolation, subsisting on very minimal alimentation (with an absence of salt, sugar, spice, alcohol, and most animal products), and also the maintenance of sexual abstinence (see also Sanz-Biset and Cañigueral, 2011; Fotiou, 2017; Gearin and Labate, 2018). One participant expressed the significance of these principles as being equally important to the plant itself:

P2: Well, for me there are two things, the conditions of the diet that are requested—the sensory isolation, the avoidance of salt, sugar et cetera, all the conditions that are required to fulfil a role so that these diet mechanisms can occur—and the plant. Mainly that, the conditions of the diet and the plants.

The status of these principles as fundamental is in line with the widely held notion “that the cure is in the diet” (“*en la dieta está la curación*”; Sanz-Biset and Cañigueral, 2011, p. 227), meaning that the healing effect is found in the whole diet complex, not simply in the intake of plants as a kind of natural pharmaceutical (Sanz-Biset and Cañigueral, 2013). Similar components making up the diet conditions were described by another informant, who also emphasized the importance of the training and knowledge of the curandero directing the diet:

P4: The diet in my view is a space in which there is a confluence firstly of isolation (the solitariness of the patient), secondly the fact of not eating any salt nor sugar, which puts the body—physical and energetic—in a very specific state, and thirdly the plant that is taken. These are the three factors, the isolation, the absence of salt and sugar, and the plant. All this creates a space in which the person in diet can reach deeply into themselves, can experience revelations and perceive things, and create a healthy space within their body and soul. The diet has to be directed by someone who knows, who is prepared, and the patient also has to be in the proper conditions to be able to cope with that diet. Basically that.

The functions of these dietary restrictions form an understanding that is multilayered: the strict food regime (sometimes near fasting) is described as affecting the physiology of the person directly, or as modulating the effect of the plant, but also as acting via its energetic functions. Correspondingly, while one participant explained the dietary restrictions in physical terms, “I think that the food restrictions are linked to the aim of preventing the body from having to do the work of digestion, of metabolizing food […] so that all the energy is dedicated to the incorporation of the plant’s energy instead” (P1), the absence of salt and sugar in the body combined with plant intake was also explained as being potentially dangerous on an energetic/spiritual level:

P2: When a person is in diet, it is like a surgery, so there are very strict conditions in order for it to work and not be dangerous—because it can be dangerous too. So dieting without salt and sugar puts you in a state of sensitivity, of vulnerability, so that the plant can be more effective; it can have effects on your body: psychological, spiritual, energetic, and physical.

Conversely, the reintroduction of salt was described as an important means of ending the diet (signalling the beginning of a post-diet phase where certain restrictions are still considered to be necessary; Sanz-Biset and Cañigueral, 2011); that is, “to cut” (*cortar*) the diet. In our interviews, the concept of the energetic body and its open or permeable state during the diet was a common means of explaining this process:

P2: You should not leave the chacra without having eaten salt, without having had a diet cut, because everything will affect you—a motorbike will pass by, you will perceive smells, you will be walking and a person who drinks alcohol may pass by—because you are very open. Once you start eating salt, your energetic body starts to close, little by little, not immediately.

#### 3.2.2 Breaking the Rules

Aside from the desirable and deliberate cutting of a diet to begin its termination process, it is also possible to accidentally cut a diet through non-observance of the diet conditions. In such cases, simply halting the effects of the plant would be the best outcome (i.e., having only cut the desired effect of the diet). In the worst case, the person is considered to have “crossed” the diet (*cruzar la dieta*), resulting in a range of possible adverse physical or psychological outcomes, which may require the intervention of a healer. For example, as the informants of Sanz-Biset and Cañigueral (2011) put it regarding the diet taboo on sexual activity:

Here sexual abstinence is of major importance. Informants declare […] with the transgression of this rule that the worst adverse reactions can happen, such as madness, or some sort of acute edema in other occasions, and that very rarely but possibly, this can lead to death. (p. 275)

Within the Amazonian medical tradition, a trained healer possesses knowledge and tools on how to remedy such situations. For example, a common technique for re-establishing the equilibrium of a person suffering from a crossed diet (*cruzadera*) is the ritualized blowing of tobacco smoke by someone trained in this technique, known as a *soplada*. This technique, widely characteristic of Peruvian Amazonian healing traditions, is considered energetic in essence (Berlowitz et al., 2020). One participant described the mechanism of a soplada in general terms, whereby the tobacco smoke functions as an “energetic bridge”, so to speak:

P7: Let’s say you have the healer’s body, and there’s the patient’s body with certain disturbances. So you make a connection with the tobacco and all those disturbances pass to the healer. The healer has to be prepared for this, to be able to assimilate, to metabolize it. […] But it’s a way to absorb things in any case, that is, the healer absorbs things from people into their body and metabolizes them. It’s as if you have a stronger tube and you can digest what the other person cannot. So you take it off, and what you can’t, you throw away, that’s why there is belching, or there are healers who have diarrhoea or even vomit for that purpose.

The idea of potentially serious adverse reactions resulting from breaking diet rules, especially the physical manifestations thereof such as skin rashes, as well as their effective resolution via energetic interventions, is widely held and applied in Amazonian medicine (Fotiou, 2017), but would not be accounted for using a psychedelic or biomedical model alone.

### 3.3 Ayahuasca in Relation to the Diet

In the survey of Sanz-Biset et al. (2008) in Chazuta, the ayahuasca vine *B. caapi* was used in the region, and 25 admixture plants were reported to be decocted with the vine in order to treat various conditions. However, there were an even wider variety of plants reported to be used in diets, with a full third of the total medicinal plants (106 of 318) being used under diet conditions, and 29 of the 106 used under diet conditions exclusively. While *B. caapi* is also potentially a diet plant, its main mode of application falls outside the scope of dietary retreats as described in this work, even though its use is still associated with alimentary and behavioural taboos. Sanz-Biset and Cañigueral's (2011) finding of diets as a primary medical intervention accords with our interview data, where it was noted that in the communities living in more remote areas of the Peruvian Amazon, diets tend to be encountered more commonly than ayahuasca:^5^

P2: In the context of the diet as it traditionally is, ayahuasca does not have any substantial role; usually there is no ayahuasca. You may find that campesinos [living in rural regions] for instance have experience with diets, but they do not drink ayahuasca or have never done so. But they do know the diet.

An overemphasis on ayahuasca as not accurately representing the overall tradition was also explicitly mentioned with respect to the international interest in Amazonian healing. One participant spoke about the importance of collecting data on the therapeutic effects of diets, “especially now, as the approach to traditional medicine by the wider public is very superficial at times, and is almost exclusively focused on ayahuasca” (P1). The perceived focus on ayahuasca is likely a function of growing Western interest and ayahuasca tourism, and the relatively large amount of academic literature directly concerning it (Beyer, 2012). As a point of comparison, although tobacco has a dominant place in Amazonian medicine (and shamanic healing beyond South America; Kroeber, 1941), there is a paucity of clinical research on it (Berlowitz et al., 2020), which is quite striking when contrasted with the body of existing work on ayahuasca (Dos Santos et al., 2016; Hamill et al., 2019). Despite this disparity in the amount of clinical literature on ayahuasca versus other aspects of traditional Amazonian medicine, the particular importance and flexibility of diets was stressed in our interviews:

P1: I think that’s what is interesting in the diets; each plant in some way responds to a particular energy that each of these plants has, and which installs itself. It’s as if the plant’s energy comes forth and installs itself in the body and structure [of the person]. It’s what I have always been saying—ayahuasca is interesting, as a visionary experience, as a thing—but it is the diet that actually goes deeper, that will break schemes and restructure. The notions that the healers have of plants is this, when they select a plant for someone, they imagine or see how this plant is—the physical plant and the effect that the plant will have on that person.

Although our interview data concerned the diets specifically, at times our participants contrasted their use with that of ayahuasca, often expressing a respect for the potency of the diet process and its ability to promote change at a deep level, either in their own experience or for others. They often tended to see a special benefit to the diet, which is in accord with the broad variety of applications that the diet is seen to have, and that we have only touched upon in this article. This view of diets contrasts with more contemporary views of Amazonian medicine that tend to focus on ayahuasca, and also points to a much larger world of Amazonian plant-based medicine.

## 4 Concluding Discussion

In traditional Amazonian medicine, the diet is a multipurpose technique that acts as a vehicle for obtaining a variety of goals, health-related and more, from a broad range of medicinal plants. The presentation of diets that we have made in this article is intended as a means of expanding the conceptual field of Amazonian medicine in the context of a resurgent psychedelic science, which features an increasing interest in indigenous plant medicines that could also be plausibly classified as “psychedelic” (cf. Ruck et al., 1979). The paradigmatic example for Amazonia has been the ayahuasca brew, and while many studies have now demonstrated its therapeutic promise (Dos Santos et al., 2016; Hamill et al., 2019; Nunes et al., 2016), it poses serious challenges with respect to scientific medical research: As with psychedelic medicine, a strictly biomedical model becomes untenable (Hartogsohn, 2017) given that the chemical effects are synergistic with at least the individual and the immediate environment, thus bringing the theoretical frame of set and setting to the fore in clinical research with ayahuasca.

However, in clinical context, set and setting are fundamentally psychopharmacological principles. Applied indiscriminately in the context of plant medicines such as ayahuasca, they may serve as a justification to dismiss the indigenous knowledge and practices around the usage of such plants as merely symbolic. However, drawing on our data and the corresponding literature presented, we suggest that experienced and trained curanderos who work with so-called “master plants” would not accept a purely symbolic interpretation of their methods—preferring instead to highlight aspects of their experience (subtle, energetic, spiritual) which they consider indispensable when using plants that induce altered states of consciousness (Andritzky, 1989; Mabit, 2007; Dobkin de Rios et al., 2008b; Berlowitz et al., 2017; Sharrock, 2018; Berlowitz et al., 2020), and pointing also to their exercise of effective techniques that can be learned using indigenous training methods (such as the diet). While it is in principle possible to reduce ayahuasca to what could be considered its “active components” (i.e., DMT and the β-carbolines)—standardizing the substance (“pharmahuasca”) and manualizing the intervention (i.e., a double reduction of the plant material and associated dynamic rituals)—such an approach could possibly even reduce therapeutic potential, not to mention threatening to marginalize the very sources of those healing practices which have so captured the attention of the West in the first place.

Yet critically, and as we have attempted to demonstrate in this article, traditional Amazonian medicine is not well represented by the use of ayahuasca alone. Any reduction to a psychological set and setting will inevitably fail to capture not only the complexities of ayahuasca healing practices, but also the broader traditions of plant medicine that make up traditional Amazonian medicine. As a medical practice, diets have a particular focus on the body and regimenting behaviour in order to achieve synergistic effects (Fotiou, 2019), and there are a large number of interacting variables that complicate the isolation of these effects. The functioning of diets is therefore poorly understood across multiple levels: from the direct physiological action of various diet plants (Sanz-Biset and Cañigueral, 2011), to depuration (Sanz-Biset and Cañigueral, 2013) and the importance of the gut (a recently emerging area in medicine and psychiatry; Foster and McVey Neufeld, 2013; Fotiou and Gearin, 2019; Mayer et al., 2015; Simpson et al., 2021), through to potentiating plant effects by regulating alimentary, sexual, and social behaviour, and including also the knowledge of healers regarding safety, indications/contraindications, adverse reactions (Berlowitz et al., 2020), and the acquisition of healing songs (Luna, 1984a; Bustos, 2008; Kaelen et al., 2018)—diets form part of a complex body of indigenous knowledge that is transmitted not only orally, but also through plant-mediated relationships (Daly and Shepard, 2019).

While the set and setting concept is quite radical in the context of a medicine that requires randomized controlled trials for establishing biomedical efficacy (Hartogsohn, 2017), it is not free of neo-colonial force when applied as a universal theory. Combined with a biomedical understanding, the concept allows for indigenous medical traditions to either have plants that work as pure biomedicine, or in the case of psychoactive plant usage, traditions that become easily relativized as “set and settings” neither culturally appropriate nor relevant to the West. The combination of isolating chemicals of interest for use in arbitrary settings leads to a decontextualized traditional medicine—one where aspects that are locally important (such as bodily techniques, and even methods of learning medicine such as the diet) are easily lost or marginalized. Antidotes to this now familiar kind of “extractive logic” can be found in developing programs of sensitive intercultural and interdisciplinary research (e.g., Sloshower, 2018), which should include collaborative ethnographic methodologies aimed to work with local ways of knowing (Hviding, 2004; Fotiou, 2019). By also incorporating other empirical research methods, tangible outcomes that are medically, psychologically, existentially/spiritually, and even ecologically significant are likely to be demonstrated, thereby contributing to a revalorization of indigenous forms of knowledge. As Hutchinson and Moerman (Hutchinson and Moerman, 2018, p. 377) write in their defence of the placebo effect,^6^ “unrefined naturalism leads to a loss of the phenomenon”; in this case, the phenomenon at hand is Amazonian medicine on its own terms as a coherent body (or more accurately, bodies) of knowledge—a medicine of the forest and land that is both traditional and contemporary.

## Data Availability

The datasets presented in this article are not readily available because the data are interviews containing personally identifiable information, and thus are not generally available for public or private access. Requests to access the datasets should be directed to ilana.berlowitz@unifr.ch.

## Footnotes

1 Chazuta is a rural region within the San Martín province; the same province in which our data was collected.

2 The specific plants used in mixes of palos/bachuja are highly variable between healers.

3 The *chacra* is a place in the forest used for dieting.

4 According to Wilbert (1991, p. 181), the practice has “almost universal distribution” in South American shamanism.

5 It should be mentioned that many South American local contexts are undergoing a restructuring as they interface with globalization, particularly as a consequence of shamanic and ayahuasca tourism (De Rios, 2005; Fotiou, 2016).

6 More precisely, the authors are defending a medically significant “meaning response” (which is likely to be validated in the context of psychedelic science; e.g., Kaelen et al., 2018).
